# Mitochondria in tumour progression: a network of mtDNA variants in different types of cancer

**DOI:** 10.1186/s12863-022-01032-2

**Published:** 2022-02-18

**Authors:** Giovanna C. Cavalcante, Ândrea Ribeiro-dos-Santos, Gilderlanio S. de Araújo

**Affiliations:** 1 Laboratory of Human and Medical Genetics, Graduate Program in Genetics and Molecular Biology, Federal University of Pará, Av. Augusto Correa, 01, Belém, PA 66075-110 Brazil; 2 Graduate Program in Oncology and Medical Sciences, Center of Oncology Research, Federal University of Pará, Rua dos Mundurucus, Belém, PA 4487, 66073-005 Brazil

**Keywords:** Cancer, Mitochondrial genome, Genetic overlap, Interactive variant networks, Oxidative stress

## Abstract

**Background:**

Mitochondrial participation in tumorigenesis and metastasis has been studied for many years, but several aspects of this mechanism remain unclear, such as the association of mitochondrial DNA (mtDNA) with different cancers. Here, based on two independent datasets, we modelled an mtDNA mutation-cancer network by systematic integrative analysis including 37 cancer types to identify the mitochondrial variants found in common among them.

**Results:**

Our network showed mtDNA associations between gastric cancer and other cancer types, particularly kidney, liver, and prostate cancers, which is suggestive of a potential role of such variants in the metastatic processes among these cancer types. A graph-based interactive web tool was made available at www2.lghm.ufpa.br/mtdna. We also highlighted that most shared variants were in the *MT-ND4*, *MT-ND5* and D-loop, and that some of these variants were nonsynonymous, indicating a special importance of these variants and regions regarding cancer progression, involving genomic and epigenomic alterations.

**Conclusions:**

This study reinforces the importance of studying mtDNA in cancer and offers new perspectives on the potential involvement of different mitochondrial variants in cancer development and metastasis.

## Background

Mitochondria are cytoplasmic organelles responsible for several pathways in cell stability and operation and are mainly known for their role in ATP production during aerobic respiration. Two of the three main steps of this process occur inside these organelles, the tricarboxylic acid cycle (TCA) in the mitochondrial matrix and oxidative phosphorylation (OXPHOS) in the mitochondrial cristae, the latter generating most of the ATP from aerobic respiration [1]. Because of their origin, mitochondria have their own genome, a double-stranded (heavy and light strands) circular molecule called the mitochondrial genome (mtgenome or mitogenome), which is highly specialized for energy metabolism. The human mtgenome is composed of 37 genes, of which 13 encode subunits of the OXPHOS protein complexes, 22 tRNA and 2 rRNA; in addition, more than 1,000 proteins encoded by the nuclear genome are necessary for overall mitochondrial function [2].

Considering the importance of mitochondrial energy metabolism and other functions to cellular homeostasis, imbalances in such processes may lead to the development of diseases. In fact, for the past few decades, mitochondria have been known to be related to cancer, but many aspects of this involvement remain unknown. Notably, the mtgenome has been reported to be altered in tumours, similar to the nuclear genome, and to be more susceptible to damage than the nuclear genome [3, 4]. Although many efforts are being made to understand the related mechanisms, it seems that mitochondrial genetics should be taken into consideration more often [5].

In this context, we recently published a study performing whole-genome sequencing for the first time in regard to gastric cancer (GC) in a Brazilian population [6]. Even more recently, a study by [7] was published with the whole mtgenome of multiple types of cancer from different populations – which do not seem to include Brazil – as part of the International Cancer Genome Consortium/The Cancer Genome Atlas Pan-Cancer Analysis of Whole Genomes Consortium (ICGC/TCGA PAWG Consortium), and the database was named The Cancer Mitochondria Atlas (TCMA). Based on these datasets, the present study aimed to analyse the mtDNA mutations that occur in common among GC and different types of cancer by modelling and developing an interactive web tool to explore the generated mtDNA mutation-cancer network, suggesting potential biomarkers in the mtgenome that may be involved in cancer development and progression.

## Results

### General analysis of the merged datasets

Here, we analysed the set of variants that are shared by individuals from two datasets, as described in the Materials and Methods section, and it should be noted that the INDEL file from the TCMA database shared no variants with the GC dataset and was therefore excluded from this study. After dataset merging, we observed 90 individuals and 22 cancer types from TCMA that presented shared variants with GC. This was probably a reflection of different genetic ancestries in both datasets, considering that the GC dataset consisted of individuals from the Brazilian Amazon region, in which Native American mtDNA haplogroups are frequently found [6], while the TCMA dataset does not seem to include the Brazilian population. This emphasizes the importance of considering the genetic background of the studied populations and stresses the potential of the shared mutations observed in this analysis.

Nevertheless, the sample size per cancer type was as follows: 7 breast adenocarcinoma (Breast-AdenoCA), 1 lobular breast cancer (Breast-LobularCA), 1 cervical squamous cell carcinoma (Cervix-SCC), 3 central nervous system medulloblastoma (CNS-Medullo), 1 central nervous system oligometastatic cancer (CNS-Oligo), 2 colorectal adenocarcinoma (ColoRect-AdenoCA), 7 oesophageal adenocarcinoma (Eso-AdenoCA), 3 head and neck squamous cell carcinoma (Head-SCC), 4 chromophobe renal cell carcinoma (Kidney-ChRCC), 9 renal cell carcinoma (Kidney-RCC), 14 hepatocellular carcinoma (Liver-HCC), 1 lung squamous cell carcinoma (Lung-SCC), 4 B-cell non-Hodgkin lymphoma (Lymph-BNHL), 2 chronic lymphocytic leukaemia (Lymph-CLL), 5 ovarian adenocarcinoma (Ovary-AdenoCA), 6 pancreatic adenocarcinoma (Panc-AdenoCA), 2 pancreatic cancer endocrine neoplasms (Panc-Endocrine), 10 prostate adenocarcinoma (Prost-AdenoCA), 1 soft tissue leiomyosarcoma (SoftTissue-Leiomyo), 1 stomach adenocarcinoma (Stomach-AdenoCA), and 3 thyroid adenocarcinoma (Thy-AdenoCA). We understand that many of these tumours have different metabolic profiles and we believe that this factor could strengthen the results found here, as shared variants in this context could represent a common pathway for cancer progression.

**Fig. 1:**
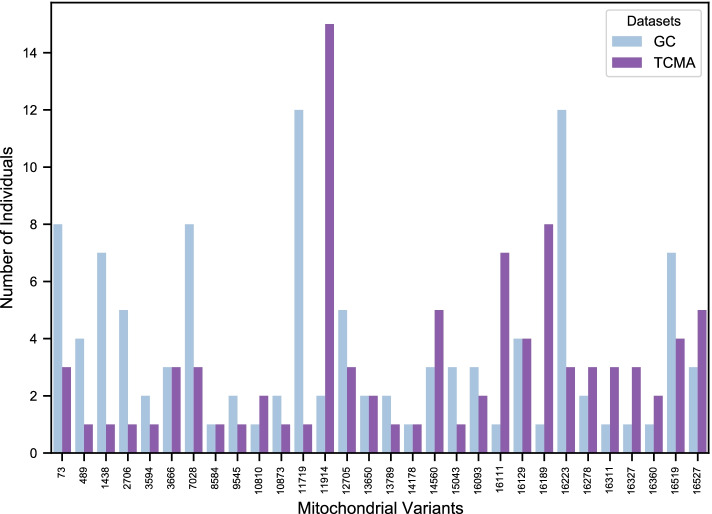
Individuals with each of the observed mitochondrial variants. Number of individuals in both datasets (GC in blue and TCMA in purple) carrying each mitochondrial variant found in the analysis

In total, 30 mitochondrial SNVs were shared between the GC and TCMA datasets, with 15 being more frequent in GC, 10 being more frequent in TCMA and five being equally frequent in these datasets (Fig. 1). All found variants were distributed in 11 mitochondrial regions: *MT-RNR1* (1438), *MT-RNR2* (2706), *T-ND1* (3594 and 3666), *MT-CO1* (7028), *MT-ATP6* (8584), *MT-CO3* (9545), *MT-ND4* (10810, 10873, 11719 and 11914), *MT-ND5* (12705, 13650 and 13789), *MT-ND6* (14178 and 14560), *MT-CYB* (15043) and D-loop (73, 489, 16093, 16111, 16129, 16189, 16223, 16278, 16311, 16327, 16360, 16519 and 16527). It is noteworthy that 11 of these variants were exclusive to tumours (somatic) in the study by [6] and distributed in four regions: D-loop (73, 489, 16093, 16189 and 16360), *MT-ND1* (3594 and 3666), *MT-ND4* (10810 and 10873), and *MT-ND5* (13650 and 13789). Regardless, from the total of 30 variants, the most frequent variants in GC were 11719 and 16223 (present in 12 individuals each), while the most frequent in TCMA was 11914 (15 individuals). Interestingly, these most frequent mutations are in either the *MT-ND4* or D-loop regions, which are also the regions containing more shared variants in this analysis.

### Shared mtDNA variants and their potential impact

**Fig. 2:**
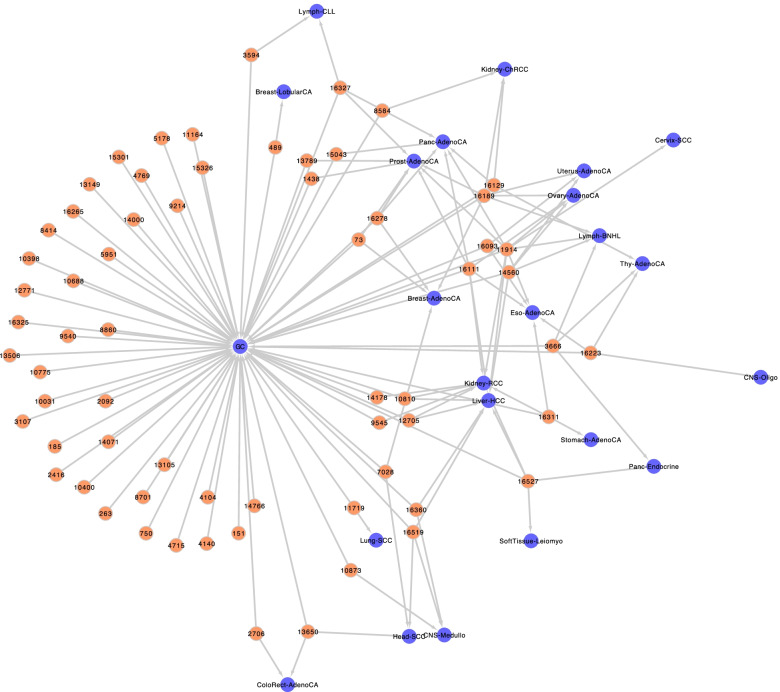
Mitochondrial mutation-cancer network. The mtDNA mutation-cancer network of mitochondrial variants (in orange) shared between different types of cancer (in blue)

Then, we performed a more in-depth analysis of the mutations in common among the different types of cancer by modelling an mtDNA mutation-cancer network, which resulted in 7,020 associations of mitochondrial variants with these 37 cancer types (36 from TCMA and GC as a reference). In Fig. 2, we plotted the mtDNA mutation-cancer network, providing a general overview of the shared variants, while Table 1 shows the statistical analysis of the variant overlaps between GC and the different cancer types, with 11 being statistically significant (from a total of 22). The empirical p-values were obtained by runs of network randomizations between pairs of cancer types regarding the Jaccard index for the number of found variants, based on the complex network strategy proposed by Araújo et al. (2016). The probability of a given Jaccard index was calculated with the equation.

**Table 1:** Statistical test summary for shared variants between GC and the other types of cancer

CT1	CT2	JC	N	P-value	Variants
GC	Breast-AdenoCA	0.006	4	***	16,189|16,278|7028|73
GC	Liver-HCC	0.007	8	***	10,810|11,914|12,705|14,560|16,111|16,360|16,519|16,527
GC	Stomach-AdenoCA	0.004	1	***	16,311
GC	Lung-SCC	0.005	1	**	11,719
GC	Prost-AdenoCA	0.011	8	**	11,914|13,789|1438|16,111|16,189|16,278|16,327|73
GC	Panc-AdenoCA	0.008	6	**	11,914|15,043|16,111|16,129|16,278|16,327
GC	Kidney-RCC	0.014	8	**	10,810|11,914|12,705|14,178|16,111|16,311|16,527|9545
GC	ColoRect-AdenoCA	0.007	2	**	13,650|2706
GC	Eso-AdenoCA	0.011	5	**	11,914|16,093|16,111|16,223|16,311
GC	Lymph-CLL	0.011	2	**	16,327|3594
GC	Kidney-ChRCC	0.011	3	**	16,129|16,189|8584
GC	Cervix-SCC	0.010	1	*	11,914
GC	Uterus-AdenoCA	0.015	3	*	14,560|16,093|16,189
GC	CNS-Medullo	0.014	3	*	10,873|16,360|16,519
GC	Ovary-AdenoCA	0.009	4	*	11,914|14,560|16,111|16,189
GC	Breast-LobularCA	0.011	1	*	489
GC	Head-SCC	0.015	3	*	13,650|16,519|7028
GC	Thy-AdenoCA	0.014	3	*	16,129|16,223|3666
GC	SoftTissue-Leiomyo	0.012	1	*	16,527
GC	CNS-Oligo	0.011	1	*	16,223
GC	Panc-Endocrine	0.009	2	*	16,527|3666
GC	Lymph-BNHL	0.016	4	*	11,914|14,560|16,189|3666

*CT *Cancer Type (1 and 2), *JC *Jaccard index, *N *Number of found variants. Statistical significance: * not significant (*P* value > 0.01); ** (*P* value < 0.01); *** (*P* value ≤ 0.000001)

$$\mathrm{P}\left({\mathrm{C}}_{\mathrm{A}}={\mathrm{SNP}}_{\mathrm{A}};{\mathrm{C}}_{\mathrm{B}}={\mathrm{SNP}}_{\mathrm{B}} \left|\mathrm{ JC}={\mathrm{C}}_{\mathrm{AB}}\right.\right)$$

where SNPA is the number of SNPs of Cancer A, SNPB is the number of SNPs of Cancer B and CAB is the JC calculated for the overlap between phenotypes A and B**.** The low values for the Jaccard index indicate that cancers showed more exclusive variants than shared ones, highlighting the found variants as potential biomarkers. Importantly, the largest number of shared mtDNA mutations per pair was eight, found in each of the following cancer types: Liver-HCC, Kidney-RCC and Prost-AdenoCA. This result suggests a possible correlation of these types with cancer progression.

Notably, 11 variants in the following regions were shared between GC and at least two other types of cancer with statistical significance: two in *MT-ND4* – 10810 (Liver-HCC and Kidney-RCC) and 11914 (Liver-HCC, Eso-AdenoCA, Kidney-RCC, Panc-Endocrine and Prost-AdenoCA); one in *MT-ND5* – 12705 (Liver-HCC and Kidney-RCC); and eight in D-loop – 73 (Breast-AdenoCA and Prost-AdenoCA), 16111 (Liver-HCC, Eso-AdenoCA, Kidney-RCC, Panc-Endocrine and Prost-AdenoCA), 16129 (Kidney-ChRCC and Panc-Endocrine), 16189 (Breast-AdenoCA, Kidney-ChRCC and Prost-AdenoCA), 16278 (Breast-AdenoCA, Panc-Endocrine and Prost-AdenoCA), 16311 (Eso-AdenoCA, Stomach-AdenoCA and Kidney-RCC), 16327 (Panc-Endocrine, Prost-AdenoCA and Lymph-CLL), and 16527 (Liver-HCC and Kidney-RCC). Curiously, there was only one variant shared with statistical significance between GC and Stomach-AdenoCA, which could be due to the reduced sample number of this type of cancer in both datasets, a general limitation of this study that could lead to a loss of information, so future studies with larger datasets are encouraged. Nevertheless, it is remarkable that all 11 variants with statistical significance in the analysed tumour samples are in *MT-ND4*, *MT-ND5* or the D D-loop, that is, two genes that encode subunits of Complex I and a regulatory region.

Among these shared variants, three are nonsynonymous, predicted as pathogenic with a Mutpred probability ranging from 0.51 to 0.63. The amino acid change associations were previously predicted between Prost-AdenoCA and *MT-ND5 (*13789 *T* > *C,* Y485H*),* Kidney-RCC and M*T-ND6 (*14178 T > C, I166V), and Kidney-ChRCC and *MT-ATP6 (*8584 G > A, A20T) [7].

### Different approaches in cancer-related SNP networks

**Fig. 3:**
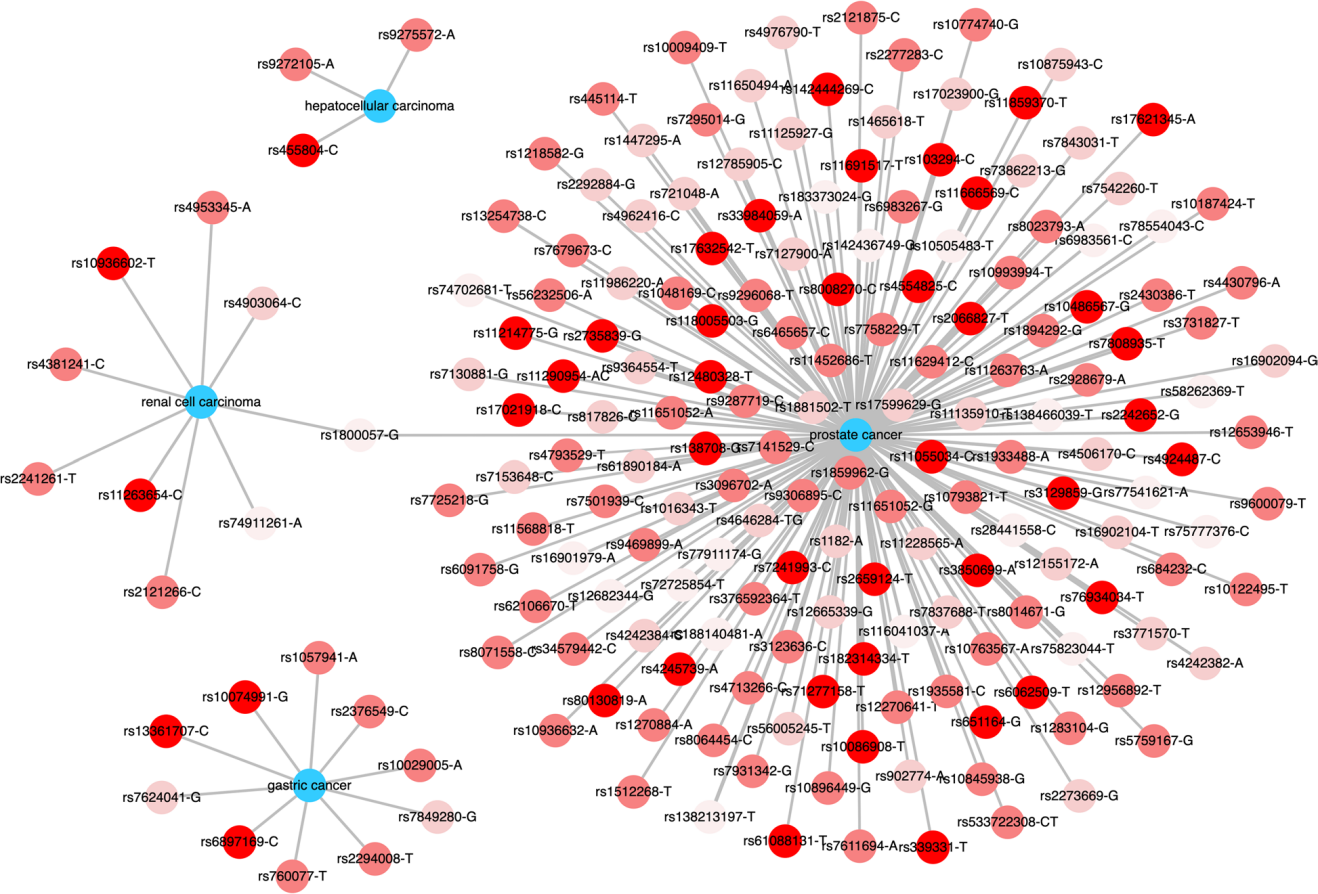
SNP-Disease Network for different types of cancer. Nuclear SNP-Disease Network for gastric cancer, renal cell carcinoma, hepatocellular carcinoma, and prostate cancer (in blue). The red gradient indicates frequency of SNPs in European populations. Low genetic overlap was found for these types of cancers at the genomic variation layer

Considering our results, as seen in Fig. 2, we stress that much information can be lost if most efforts only focus on the nuclear genome, leaving the mitochondrial genome aside. Indeed, by dissecting the mtDNA network, we identified genetic overlaps suggesting novel events that are not observed when building nuclear SNP-disease networks. In Fig. 3, we show a network of this sort that was modelled using GWAS hits from the DANCE web tool regarding GC, prostate cancer, renal cell carcinoma and hepatocellular carcinoma. This finding adds a layer of complexity in understanding the genetics of several cancers. In this context, our study contributes to the identification of a set of mitochondrial variants associated with GC and other types of cancer.

Taking this into consideration, we developed a graph-based visualization tool of the dataset overlaps analysed here to improve the understanding of the relationship among mtDNA variants and cancer types. The mtDNA mutation-cancer interactions found here were catalogued and reported as seen in Fig. 2, along with raw data for the network, which can be interactively explored. This user-friendly web tool was made freely available at https://www2.lghm.ufpa.br/mtdna.

## Discussion

In this study, our analyses with multiple types of cancer have highlighted different mtDNA mutations, particularly in the *MT-ND4*, *MT-ND5* and D-loop, suggesting a special importance of such mitochondrial mutations and regions during cancer development and progression. The *MT-ND4* and *MT-ND5* genes encode core subunits of respiratory chain complex I (also known as NADH dehydrogenase), which receives electrons from carrier NADH (reduced nicotinamide adenine dinucleotide) after the TCA cycle, initiating the process of OXPHOS [1]. Because OXPHOS is responsible for most ATP produced in aerobic cellular respiration and Complex I is the main entry of electrons, defects in this complex could result in impairment of the entire process. It can also increase the production of reactive oxygen species (ROS), culminating in oxidative stress and promoting tumorigenesis [8]. In fact, mutations in *MT-ND4* and *MT-ND5* leading to complex I dysfunction have been previously associated with procancerous phenotypes and tumorigenesis in different types of cancer, although the particularities of this relationship are still unknown [9, 10].

The D-loop, which is part of the major noncoding region (NCR) in the mt genome, is responsible for the start of heavy strand replication, as well as the transcription of both strands, and it is known to be hypervariable [11, 12]. Since D-loop and NCR are terms frequently used as synonyms, it should be noted that, here, "D-loop” represents not only the D-loop per se but also the major NCR. Nevertheless, abnormalities in replication and transcription of mtDNA could lead to mitochondrial instability and malfunction, which in turn could play a role in carcinogenesis and metastasis. Independent studies have reported D-loop variants in association with different kinds of cancer, including gastric cancer, but there is still no consensus as to which variants influence this process [12–14].

Notably, epigenomic factors may also play a role in D-loop function. For instance, long noncoding RNAs (lncRNAs) generated by heavy strand transcription have been described in the D-loop with an interaction with mitochondrial topoisomerase 1B (TOP1MT), which normally promotes mtDNA replication, suggesting a possible influence of these lncRNAs on the regulation of mtDNA expression [15]. In addition, treatment with miR34a encapsulated in hyaluronic acid nanoparticles has been demonstrated to reduce CpG methylation in the D-loop and to increase mRNA transcripts of different mitochondrial genes in lung cancer cells [16]. Currently, little is known about epigenomic mechanisms such as noncoding RNAs in mitochondria, particularly in D-loops, but it is increasingly clear that this matter should be further explored [1].

Regardless, in our analyses, it was also notable that 11914 (*MT-ND4*) and 16111 (D-loop) are not only present in the greatest number of cancer types, but these types are the same (hepatocellular carcinoma, oesophageal adenocarcinoma, pancreatic adenocarcinoma, renal cell carcinoma and prostate adenocarcinoma). Additionally, 10810 (*MT-ND4*), 12705 (*MT-ND5*) and 16527 (D-loop) are also shared by the same cancer types (hepatocellular carcinoma and renal cell carcinoma). Another interesting finding, as previously mentioned, is that kidney, prostate, and liver cancers (renal cell carcinoma, prostate adenocarcinoma, and hepatocellular carcinoma, respectively) presented more shared variants with GC than the other types of cancer (eight variants).

Curiously, no studies were found reporting a positive association with such variants in cancer, except for 16189 (D-loop). This mutation, which is suggested to generate mitochondrial impairment and genome instability, has been related to different complex conditions, such as endometriosis [17], coronary artery disease [18], type 2 diabetes mellitus and metabolic syndrome [19], as well as multiple types of cancer, namely, endometrial [20], breast [21], colorectal [22] and acute myeloid leukaemia [23]. Additionally, a study by [24] suggested that variant 16189 could especially affect iron homeostasis and electron transport chains of OXPHOS, leading to increased oxidative stress. In fact, excess iron has been considered a risk factor for cancer development and progression [25–27].

Notably, mitochondria have been associated with tumorigenesis and metastasis, even though the mitochondrial mechanisms and dynamics involved in these processes are not fully understood [28]. A recent review by [29] highlighted the importance of studying mitochondrial dysfunction in different complex diseases, including cancer, in regard to mtDNA alterations as potential biomarkers for the development, metastasis and prognosis of cancer. In this sense, our findings might shed light on this matter, considering the overlap of cancer types, particularly GC with kidney, liver, and prostate cancers, which allowed us to hypothesize that these variants are likely to play a role in their progression with potential involvement in a metastatic process between these types of cancer.

In the specialized literature, although considered an uncommon process, it is possible to find various case reports of gastric metastases from renal cell carcinoma [30, 31] or prostatic adenocarcinoma [32, 33], even several years after the first diagnosis [34]. A secondary tumour might also be so similar to a primary tumour that it could be challenging to diagnose it as a metastatic tumour at first [35]. Recently, a case report has reinforced that it is still unclear how gastric metastasis from prostate cancer occurs – whether it is by lymphatic or haematogenous spread – as well as the importance of an early diagnosis [36]. It is noteworthy that some cases of prostate or kidney metastasis from gastric adenocarcinoma have also been reported [37, 38]. Regarding liver metastasis from gastric cancer, it has been pointed out that the liver is one of the most frequently affected organs in metastasis from gastric cancer: metastasis is found in approximately 35% of gastric cancer cases at first diagnosis, of which up to 14% involve this organ [39, 40]. On the other hand, hepatocellular carcinoma with gastric metastasis seems to be very rare, occurring in less than 1% of patients with this type of cancer, and thus, it is possible to misdiagnose it as gastric cancer with liver metastasis [41].

In this context, understanding the effects of genetic variants on complex diseases such as cancer may directly impact clinical strategies for personalized patient care. Approaches to building disease networks have been previously developed based on genetic associations, mainly using autosomal DNA, molecular pathways or candidate nuclear genes [42, 43]. Genetic comorbidity was shown for intraclass diseases, such as neuropsychiatric disorders, and this was recently found for cancer in a TCGA (The Cancer Genome Atlas) analysis that reported 10 signalling pathways in common among 33 tumours [44]. The comprehension of interactions between phenotype and genotype and between intraclass diseases impacts the understanding of many progressive aspects of a disease such as cancer, including cancer types, metastatic mechanisms and the development of a personalized diagnosis or prognosis, as well as pharmacogenomic prevention strategies.

Importantly, genetic studies have not generally considered mitochondrial variants on a large scale and their impact during investigations of genetic comorbidity. Here, we have shown that many variants of interest could be overlooked, so we highlight the idea of data integration with several layers of genetic associations for modelling disease networks. This approach may contribute to the identification of patterns of genetic similarity and to the discovery of new mtDNA associations in the mechanism of gastric cancer and other types of cancer.

## Conclusions

In summary, this work has provided new information on the mitochondrial influence on tumorigenesis and metastasis from a network perspective, indicating some potential mtDNA biomarkers – particularly *MT-ND4*, *MT-ND5* and D-loop – and emphasizing the importance of studying mitochondrial genetics in association with cancer progression. Therefore, considering the sample number to be a limitation of this work (together with the absence of heteroplasmy and haplogroup analyses due to a lack of data), future studies with larger datasets in systems biology, as well as functional studies, are recommended to reinforce the observed interactions of these variants in different types of cancer. We also provide a user-friendly web tool to explore the broad set of shared mitochondrial variants among cancers that may support larger datasets from future studies, encouraging the development of new software for cancer and genetics epidemiology while also improving the transparency and reproducibility of research studies.

## Methods

### Data Collection and Characterization

Data of mitochondrial variants were extracted from two sources: (i) homoplasmic mtDNA mutations in gastric cancer from the study by [6] and (ii) the mutation section (Single Nucleotide Variant – SNV and Insertion/Deletion – INDEL files) of The Cancer Mitochondria Atlas (TCMA) portal (https://ibl.mdanderson.org/tcma/), which was made public and freely available by [7]. The first dataset included 13 tumour samples from gastric cancer (GC) patients, while the second included 2,536 tumour samples distributed in 36 cancer types. More detailed characterization of the samples can be found in the mentioned works.

### Data Analyses

Datasets from both sources were merged into one, considering each variant/position in common. For that and all other analyses, we used Python 3 programming language and the following libraries in this environment: Pandas [45], Matplotlib [46], Seaborn [47] and Scipy [48].

### The mtDNA mutation-cancer network

We constructed a bipartite network of mitochondrial mutations and cancer with datasets of different types of cancer. The mtDNA mutation-cancer network is presented as a graph G(V, E), where V comprises two distinct sets of nodes: mitochondrial mutations (u) and cancers (v). In this analysis, we considered a mitochondrial mutation to be associated with cancer if this association was reported in the works that originated the analysed datasets [6, 7].

Next, we assessed the topology of the mtDNA mutation-cancer network and computed an empirical p value of mutation overlap (i.e., mutations in common) between pairs of cancer types. This was done by runs of 100 K randomization of associations between the cancers to generate their empirical distribution. Thus, the probability (P) of a given overlap was calculated with P(NA, NB | NAB), where NA is the number of mitochondrial mutations of cancer "A", NB is the number of mitochondrial mutations of cancer "B" and NAB is the overlap calculated for the pair of cancers "A" and "B". This strategy was inspired by the work of [42], which found significant association overlap for complex diseases based on data from the NHGRI/EBI GWAS Catalog (https://www.ebi.ac.uk/gwas/).

We then implemented a web tool to better visualize and explore this mtDNA mutation-cancer network. HTML5 with the bootstrap framework (https://getbootstrap.com/) was used for front-end development. The mtDNA mutation-cancer network was generated using the D3.js library, and raw data were implemented with DataTables (https://datatables.net/), with both libraries in JavaScript and JQuery (https://jquery.com/). The network and raw data were stored in JSON files and accessed via Ajax combined with libraries in Python 3 using the Flask framework for web applications (https://flask.palletsprojects.com/en/1.1.x/) in the backend.

### Nuclear SNP-Disease Network

We retrieved genetic association data using the Disease-Ancestry Network (DANCE – www.ldgh.com.br/dance), which is a web tool that allows the recovery of associations among complex diseases and nuclear single nucleotide polymorphisms (SNPs) in a bipartite network called SNP-Disease Network [42]. DANCE integrates data from the 1000 Genomes Project (Phase 3) [49] and the NHGRI GWAS Catalog (v. March 2020) [50], comprising data from 3,885 public genome-wide association studies (GWAS). DANCE database stores 149,262 associations among 4,208 phenotypes and 120,748 risk-alleles. We queried risk-alleles associations with GC, renal cell carcinoma, hepatocellular carcinoma, and prostate cancer. Risk-alleles were considered for those variants that reached a statistical significance (*P*-value ≤ 1e-8) and odds ratio > 1.

## Data Availability

The datasets generated and/or analysed during the current study are available in the FigShare repository (https://doi.org/10.6084/m9.figshare.15062628).

## Abbreviations

ATP: Adenosine triphosphate
Breast-AdenoCA: Breast adenocarcinoma
Breast-LobularCA: Lobular breast cancer
Cervix-SCC: Cervical squamous cell carcinoma
CNS-Medullo: Central nervous system medulloblastoma
CNS-Oligo: central nervous system oligometastatic cancer
ColoRect-AdenoCA: Colorectal adenocarcinoma
CT1: Cancer type 1
CT2: Cancer type 2
D-loop: Displacement loop
DANCE: Disease-Ancestry Network
EBI: European Bioinformatics Institute
Eso-AdenoCA: Esophageal adenocarcinoma
GC: Gastric cancer
GWAS: Genome-wide analysis study
Head-SCC: Head and neck squamous cell carcinoma
ICGC/TCGA PAWG Consortium: International Cancer Genome Consortium/The Cancer Genome Atlas Pan-Cancer Analysis of Whole Genomes Consortium
INDEL: Insertion/Deletion
JC: Jaccard index
Kidney-ChRCC: Chromophobe renal cell carcinoma
Kidney-RCC: Renal cell carcinoma
Liver-HCC: Hepatocellular carcinoma
lncRNAs: Long non-coding RNAs
Lung-SCC: Lung squamous cell carcinoma
Lymph-BNHL: B-cell non-Hodgkin lymphoma
Lymph-CLL: Chronic lymphocytic leukemia
miR34a: MicroRNA 34a
mRNA: Messenger RNA
MT-ATP6: Mitochondrially encoded ATP synthase 6, also known as ATP6
MT-CO1: Mitochondrially encoded cytochrome c oxidase I, also known as CO1
MT-CO3: Mitochondrially encoded cytochrome c oxidase III, also known as CO3
MT-CYB: Mitochondrially encoded cytochrome b, also known as CYB
MT-ND1: Mitochondrially encoded NADH dehydrogenase 1, also known as ND1
MT-ND4: Mitochondrially encoded NADH dehydrogenase 4, also known as ND4
MT-ND5: Mitochondrially encoded NADH dehydrogenase 5, also known as ND5
MT-ND6: Mitochondrially encoded NADH dehydrogenase 6, also known as ND6
MT-RNR1: Mitochondrially encoded 12S RNA, also known as RNR1
MT-RNR2: Mitochondrially encoded 16S RNA, also known as RNR2
mtDNA: Mitochondrial DNA
mtgenome: Mitochondrial genome
N: Number of found variants
NA: The number of mitochondrial mutations of cancer "A" in P(NA, NB | NAB)
NAB: The overlap calculated for the pair of cancers "A" and "B" in P(NA, NB | NAB)
NADH: Reduced nicotinamide adenine dinucleotide or nicotinamide adenine dinucleotide (NAD) + hydrogen (H)
NB: The number of mitochondrial mutations of cancer "B" in P(NA, NB | NAB)
NCR: Non-coding region
NHGRI: National Human Genome Research Institute
Ovary-AdenoCA: Ovarian adenocarcinoma
OXPHOS: Oxidative phosphorylation
P: Probability of a given overlap, calculated with P(NA, NB | NAB)
Panc-AdenoCA: Pancreatic adenocarcinoma
Panc-Endocrine: Pancreatic cancer endocrine neoplasms
Prost-AdenoCA: Prostate adenocarcinoma
ROS: Reactive oxygen species
rRNA: Ribosomal RNA
SNP: Single nucleotide polymorphism
SNV: Single nucleotide variant
SoftTissue-Leiomyo: Soft tissue leiomyosarcoma
Stomach-AdenoCA: Stomach adenocarcinoma
TCA: Tricarboxylic acid cycle
TCGA: The Cancer Genome Atlas
TCMA: The Cancer Mitochondria Atlas
Thy-AdenoCA: Thyroid adenocarcinoma
TOP1MT: Mitochondrial topoisomerase 1B
tRNA: Transfer RNA
Uterus-AdenoCA: Uterus adenocarcinoma

## References

[1] Cavalcante GC, Magalhães L, Ribeiro-Dos-Santos Â, Vidal AF (2020). Mitochondrial Epigenetics: Non-Coding RNAs as a Novel Layer of Complexity. Int J Mol Sci.

[2] Wei W, Chinnery PF (2020). Inheritance of mitochondrial DNA in humans: implications for rare and common diseases. J Intern Med.

[3] Keogh MJ, Chinnery PF (2015). Mitochondrial DNA mutations in neurodegeneration. Biochimica et Biophysica Acta (BBA) Bioenergetics.

[4] Weigl S, Paradiso A, Tommasi S (2013). Mitochondria and Familial Predisposition to Breast Cancer. Curr Genomics.

[5] Gammage PA, Frezza C (2019). Mitochondrial DNA: the overlooked oncogenome?. BMC Biol.

[6] Cavalcante GC, Marinho ANR, Anaissi AK, Vinasco-Sandoval T, Ribeiro-Dos-Santos A, Vidal AF (2019). Whole mitochondrial genome sequencing highlights mitochondrial impact in gastric cancer. Sci Rep.

[7] Yuan Y, Ju YS, Kim Y, Li J, Wang Y, Yoon CJ (2020). Comprehensive molecular characterization of mitochondrial genomes in human cancers. Nat Genet.

[8] Saikolappan S, Kumar B, Shishodia G, Koul S, Koul HK (2019). Reactive oxygen species and cancer: A complex interaction. Cancer Lett.

[9] Singh RK, Saini S, Verma D, Kalaiarasan P, Bamezai RNK (2017). Mitochondrial ND5 mutation mediated elevated ROS regulates apoptotic pathway epigenetically in a P53 dependent manner for generating pro-cancerous phenotypes. Mitochondrion.

[10] Tzen C-Y, Mau B-L, Wu T-Y (2007). ND4 mutation in transitional cell carcinoma: Does mitochondrial mutation occur before tumorigenesis?. Mitochondrion.

[11] Nicholls TJ, Minczuk M (2014). In D-loop: 40 years of mitochondrial 7S DNA. Exp Gerontol.

[12] Zhao Y-B (2005). Mutation in D-loop region of mitochondrial DNA in gastric cancer and its significance. WJG.

[13] Wang H, Wang Y, Zhao Q, Guo Z, Zhang F, Zhao Y (2016). Identification of sequence polymorphisms in the D-Loop region of mitochondrial DNA as a risk factor for gastric cancer. Mitochondrial DNA A DNA Mapp Seq Anal.

[14] Wei L, Zhao Y, Guo T, Li P, Wu H, Xie H (2011). Association of mtDNA D-Loop Polymorphisms with Risk of Gastric Cancer in Chinese Population. Pathology & Oncology Research.

[15] Dalla Rosa I, Zhang H, Khiati S, Wu X, Pommier Y (2017). Transcription profiling suggests that mitochondrial topoisomerase IB acts as a topological barrier and regulator of mitochondrial DNA transcription. J Biol Chem.

[16] Trivedi M, Singh A, Talekar M, Pawar G, Shah P, Amiji M (2017). MicroRNA-34a Encapsulated in Hyaluronic Acid Nanoparticles Induces Epigenetic Changes with Altered Mitochondrial Bioenergetics and Apoptosis in Non-Small-Cell Lung Cancer Cells. Sci Rep.

[17] Cho S, Lee Y-M, Choi YS, Yang HI, Jeon YE, Lee KE (2012). Mitochondria DNA polymorphisms are associated with susceptibility to endometriosis. DNA Cell Biol.

[18] Mueller EE, Eder W, Ebner S, Schwaiger E, Santic D, Kreindl T, et al. The mitochondrial T16189C polymorphism is associated with coronary artery disease in Middle European populations. PLoS ONE. 2011;6:e16455. 10.1371/journal.pone.0016455 PMC3027676 21298061

[19] Kwak SH, Park KS (2016). Role of mitochondrial DNA variation in the pathogenesis of diabetes mellitus. Front Biosci (Landmark Ed).

[20] Liu VWS, Yang HJ, Wang Y, Tsang PCK, Cheung ANY, Chiu PM (2003). High frequency of mitochondrial genome instability in human endometrial carcinomas. Br J Cancer.

[21] Tipirisetti NR, Govatati S, Pullari P, Malempati S, Thupurani MK, Perugu S, et al. Mitochondrial control region alterations and breast cancer risk: a study in South Indian population. PLoS ONE. 2014;9:e85363. 10.1371/journal.pone.0085363 PMC3907410 24497926

[22] Govatati S, Saradamma B, Malempati S, Dasi D, Thupurani MK, Nagesh N (2017). Association of mitochondrial displacement loop polymorphisms with risk of colorectal cancer in south Indian population. Mitochondrial DNA A DNA Mapp Seq Anal.

[23] Kim HR, Kang M-G, Lee YE, Na BR, Noh MS, Yang SH (2018). Spectrum of mitochondrial genome instability and implication of mitochondrial haplogroups in Korean patients with acute myeloid leukemia. Blood research.

[24] Salvador M, Villegas A, Llorente L, Ropero P, González FA, Bustamante L (2007). 16189 Mitochondrial variant and iron overload. Ann Hematol.

[25] Cheng M, Liu P, Xu LX (2020). Iron promotes breast cancer cell migration via IL-6/JAK2/STAT3 signaling pathways in a paracrine or autocrine IL-6-rich inflammatory environment. J Inorg Biochem.

[26] Khan A, Singh P, Srivastava A (2020). Iron: Key player in cancer and cell cycle?. J Trace Elem Med Biol.

[27] Manz DH, Blanchette NL, Paul BT, Torti FM, Torti SV (2016). Iron and cancer: recent insights. Ann N Y Acad Sci.

[28] Denisenko TV, Gorbunova AS, Zhivotovsky B (2019). Mitochondrial Involvement in Migration, Invasion and Metastasis. Front Cell Dev Biol.

[29] Nguyen NNY, Kim SS, Jo YH (2020). Deregulated Mitochondrial DNA in Diseases. DNA Cell Biol.

[30] O’Reilly MK, Sugrue G, Han-Suyin K, Fenlon H (2017). Radiological, pathological and gross correlation of an isolated renal cell carcinoma metastasis to the stomach. BMJ Case Rep.

[31] Pollheimer MJ, Hinterleitner TA, Pollheimer VS, Schlemmer A, Langner C (2008). Renal cell carcinoma metastatic to the stomach: single-centre experience and literature review. BJU Int.

[32] Bilici A, Dikilitas M, Eryilmaz OT, Bagli BS, Selcukbiricik F (2012). Stomach metastasis in a patient with prostate cancer 4 years after the initial diagnosis: a case report and a literature review. Case Rep Oncol Med.

[33] Solis Lara HE, Villarreal Del Bosque N, SadaTreviño MA, Yamamoto Ramos M, Argueta Ruiz RDC (2018). Gastric Metastasis of Prostate Cancer as an Unusual Presentation Using 68Ga-Prostate-Specific Membrane Antigen PET/CT. Clin Nucl Med.

[34] Namikawa T, Iwabu J, Kitagawa H, Okabayashi T, Kobayashi M, Hanazaki K (2012). Solitary gastric metastasis from a renal cell carcinoma presenting 23 years after radical nephrectomy. Endoscopy.

[35] Inagaki C, Suzuki T, Kitagawa Y, Hara T, Yamaguchi T (2017). A case report of prostate cancer metastasis to the stomach resembling undifferentiated-type early gastric cancer. BMC Gastroenterol.

[36] Then EO, Nutakki S, Ofosu A, Saleem S, Saleem V, Sunkara T (2020). An Unlikely Culprit: Gastric Metastasis from Primary Prostatic Adenocarcinoma. J Gastrointest Cancer.

[37] Wada Y, Yoshida K, Hihara J, Tanabe K, Ukon K (2007). Kidney metastasis of resected early gastric carcinoma: report of a case. Surg Today.

[38] Zhang P, Zheng Y, Ran H, Leng Z, Wang Z (2010). Case report: gastric adenocarcinoma metastatic to the prostate gland. J Radiol Case Rep.

[39] Uggeri F, Ripamonti L, Pinotti E, Scotti MA, Famularo S, Garancini M (2020). Is there a role for treatment-oriented surgery in liver metastases from gastric cancer?. World J Clin Oncol.

[40] Zacherl J, Zacherl M, Scheuba C, Steininger R, Wenzl E, Mühlbacher F (2002). Analysis of hepatic resection of metastasis originating from gastric adenocarcinoma. J Gastrointest Surg.

[41] Peng L, Yu K, Li Y, Xiao W (2018). Gastric metastasis of recurrent hepatocellular carcinoma: A case report and literature review. J Cancer Res Ther.

[42] Araújo GS, Lima LHC, Schneider S, Leal TP, da Silva APC, Vaz de Melo POS (2016). Integrating, summarizing and visualizing GWAS-hits and human diversity with DANCE (Disease-ANCEstry networks). Bioinformatics.

[43] Goh K-I, Cusick ME, Valle D, Childs B, Vidal M, Barabási A-L (2007). The human disease network. Proc Natl Acad Sci USA.

[44] Sanchez-Vega F, Mina M, Armenia J, Chatila WK, Luna A, La KC (2018). Oncogenic Signaling Pathways in The Cancer Genome Atlas. Cell.

[45] Reback J, McKinney W, Mendel B, Van den Bossche J, Augspurger T, Cloud P, et al. pandas-dev/pandas: Pandas 1.0.3. Zenodo; 2020. 10.5281/zenodo.3715232.

[46] Hunter JD (2007). Matplotlib: A 2D Graphics Environment. Computing in Science Engineering.

[47] Waskom M, Botvinnik O, O’Kane D, Hobson P, Lukauskas S, Gemperline DC, et al. mwaskom/seaborn: v0.8.1 (September 2017). Zenodo; 2017. 10.5281/zenodo.883859.

[48] Virtanen P, Gommers R, Oliphant TE, Haberland M, Reddy T, Cournapeau D (2020). SciPy 1.0: fundamental algorithms for scientific computing in Python. Nat Methods.

[49] Auton A, Brooks LD, Durbin RM, Garrison EP, Kang HM, 1000 Genomes Project Consortium (2015). A global reference for human genetic variation. Nature.

[50] Buniello A, MacArthur JAL, Cerezo M, Harris LW, Hayhurst J, Malangone C (2019). The NHGRI-EBI GWAS Catalog of published genome-wide association studies, targeted arrays and summary statistics 2019. Nucleic Acids Res.

